# The Potential Anti-Photoaging Effect of Photodynamic Therapy Using Chlorin e6-Curcumin Conjugate in UVB-Irradiated Fibroblasts and Hairless Mice

**DOI:** 10.3390/pharmaceutics14050968

**Published:** 2022-04-30

**Authors:** Ga-Hee Hur, A-Reum Ryu, Yong-Wan Kim, Mi-Young Lee

**Affiliations:** 1 Department of Medical Sciences, Soonchunhyang University, Asan 31538, Korea; gjrkgmlsid@naver.com; 2 Department of Medical Biotechnology, Soonchunhyang University, Asan 31538, Korea; yar4310@sch.ac.kr; 3 Dongsung Bio Pharmaceutical Co., Ltd., Seoul 01340, Korea; thomas06@hanmail.net

**Keywords:** photodynamic therapy, chlorin e6, PEG, curcumin, UVB, wrinkle, collagen

## Abstract

Photodynamic therapy (PDT) has been used to treat cancers and non-malignant skin diseases. In this study, a chlorin e6–curcumin conjugate (Ce6-PEG-Cur), a combination of chlorin e6 (Ce6) and curcumin via a PEG linker, was used as a photosensitizer. The in vitro and in vivo effects of PDT using Ce6-PEG-Cur were analyzed in UVB-irradiated fibroblasts and hairless mice. The UVB-induced expression of MMPs was reduced in Hs68 fibroblast cells, and procollagen type Ⅰ expression was enhanced by Ce6-PEG-Cur-mediated PDT on a Western blotting gel. Moreover, UVB-induced collagen levels were restored upon application of Ce6-PEG-Cur-mediated PDT. Ce6-PEG-Cur-mediated PDT inhibited the expression of phosphorylated p38 in the MAPK signaling pathway, and it reduced the expression of phosphorylated NF-κB. In animal models, Ce6-PEG-Cur-mediated PDT inhibited the expression of MMPs, whereas procollagen type Ⅰ levels were enhanced in the dorsal skin of UVB-irradiated mice. Moreover, UVB-induced dorsal roughness was significantly reduced following Ce6-PEG-Cur-mediated PDT treatment. H&E staining and Masson’s trichrome staining showed that the thickness of the epidermal region was reduced, and the density of collagen fibers increased. Taken together, Ce6-PEG-Cur-mediated PDT might delay and improve skin photoaging by ultraviolet light, suggesting its potential for use as a more effective photo-aging treatment.

## 1. Introduction

Skin aging can be categorized into intrinsic aging (chronologic aging) and extrinsic aging (photoaging) [1,2]. Intrinsic skin aging is a natural consequence of biological change. Intrinsically aged skin appears smooth, pale, and finely wrinkled. In contrast, extrinsically aged skin is coarsely wrinkled and is frequently characterized by abnormal pigmentation, epidermal hyperplasia, degraded collagen fibrils, and an accumulation of irregular elastic fibers [3,4]. Extrinsic skin aging is caused by various environmental factors, such as UV exposure, pollution, and different lifestyle components [5].

Skin photoaging is related to the generation of reactive oxygen species (ROS) caused by UV exposure [6]. Ultraviolet-induced ROS can lead to skin damage, inflammation, and skin cancer. Enhanced matrix metalloproteinase (MMP) expression induced by ROS can cause collagen degradation in the extracellular matrix (ECM) [7], leading to the skin wrinkling that characterizes aged skin [8].

ROS generation by UVB irradiation can induce MMP expression by activating transcription factors, such as nuclear factor-kappa B (NF-κB) and activator protein-1 (AP-1) [9,10]. The presence of ROS induces the mitogen-activated protein (MAP) kinase protein, extracellular signal-regulated kinase (ERK), p38, and c-Jun N-terminal kinase (JNK). Activated MAPKs are translocated to the nucleus, and the transcription factor AP-1 promotes MMP expression [11,12]. Besides AP-1, NF-κB is another important transcription factor that is activated in response to UV irradiation, which regulates inflammatory mediators such as iNOS and COX-2 [13,14]. Therefore, the inhibition of NF-κB activation and the AP-1 pathway would interrupt the biological pathway that leads to the chronic inflammation and skin aging induced by UV irradiation [15].

MMPs are zinc-dependent endopeptidases involved in the remodeling of the extracellular matrix, which play various roles in morphogenesis, angiogenesis, arthritis, and tumor metastasis [16,17]. UV radiation can elevate the levels of various MMPs, including MMP-1, MMP-2, and MMP-9, and degrade the ECM, resulting in wrinkle formation and increased skin thickness in humans [18]. MMP-1, a well-known interstitial collagenase, is primarily responsible for the digestion of type Ⅰ collagen, which is the most abundant in skin connective tissue [19]. MMP-2 and -9, which belong to the gelatinase MMPs, degrade the ECM in the basement membrane and elastin by degrading type Ⅳ collagen [20].

Photodynamic therapy (PDT), involves a photosensitizer, light source, and molecular oxygen [21], and it has been used for the treatment of a variety of dermatological disorders and cancers [22,23]. Recently, the use of light-emitting diodes (LEDs) has led to the clinical application of PDT for a variety of medical and cosmetic needs [24]. The advantage of LED light is that it is less invasive and has fewer side effects in targeted tissues than lasers [25]. Moreover, the adverse effects of PDT can be minimized by adjusting the dosage of the photosensitizer and the intensity of the light source [26].

Chlorin e6 (Ce6), a second generation photosensitizer, is known for its anti-microbial, anti-fungal [27], anti-oxidative [28], anti-wrinkle [29], anti-sebostatic [30], anti-inflammatory [31], and anti-obesity [32] activities in PDT, as reported in our previous papers. Moreover, the Ce6 and curcumin conjugate (Ce6-PEG-Cur) showed a remarkable capability to trigger intrinsic apoptosis in pancreatic cell lines [33]; however, information on the effect of a Ce6-Cur conjugate on the skin is not currently available. The purpose of this study was to investigate the efficacy of PDT with Ce6-PEG-Cur on UVB-induced skin photoaging. The in vitro and in vivo inhibitory effects of Ce6-PEG-Cur-mediated PDT on UVB-induced wrinkle formation were examined in this study.

## 2. Materials and Methods

### 2.1. Ce6-PEG-Cur Synthesis

The Ce6-cur conjugate was synthesized by incorporating a 2,2′-(ethylenedioxy)-bis-(ethylamine) (mono PEG) linker using the methods reported in a previous study [33]. Di-tert-butyldicarbonate was added to a solution of 2,2′-(ethylenedioxy)-bis-(ethylamine) in anhydrous CHCl_3_ at 0 °C to obtain tert-butyl-(2-(2-(2-aminoethoxy)ethoxy)ethyl)carbamate. The 5-(4-((1E, 6E)-7-(4-hydroxy-3-methoxyphenyl)-3, 5-dioxohepta-1, 6-dien-1-yl)-2-methoxyphenoxy)-5-oxopentanoic acid was prepared by the reaction of curcumin, DMAP, Et_3_N, and glutaric anhydride in THF under reflux conditions. Furthermore, the dimethyl ester of Ce6 was prepared by reacting Ce6 with 5% sulfuric acid in methanol at room temperature. The DME Ce6 thus obtained was treated with EDCI, HOBt tert-butyl(2-(2-(2-aminoethoxy)ethoxy)ethyl)carbamate, and DIEA to obtain Ce6-MonoPEG-NHBoc at room temperature. This compound was de-protected by reacting with TFA to obtain Ce6-MonoPEGamine. For the synthesis of the Ce6-PEG-curcumin conjugate, HOBt, EDCI, and DIEA were dissolved in dry CH_2_Cl_2_ then stirred for 30 min. DIPEA was added followed by overnight stirring at room temperature. The reaction was quenched with 5% aqueous citric acid. Silica gel column chromatography was utilized to afford Ce6-PEG-curcumin.

### 2.2. In Vitro Assay

#### 2.2.1. Determination of the Antioxidant Capacity of Ce6-PEG-Cur

The antioxidant activity of Ce6-PEG-Cur was determined using 2,2′-azino-bis(3-ethylbenzothiazoline-6-sulfonic acid) (ABTS^•+^) radical cation-based assays and the oxygen radical absorbance capacity (ORAC) assay three times for reproducibility. The ABTS radical scavenging capacity of Ce6-PEG-Cur was determined according to a modified version of the method described by [34]. The ORAC assay is based on the scavenging of peroxyl radicals generated by 2,2′-azobis(2-methylpropioamidine) dihydrochloride (AAPH), which prevents the degradation of the fluorescein probe. The ORAC assay for Ce6-PEG-Cur (10 μM) was performed according to a modified version of the method of Ou et al. (2001) [35]. The anti-radical capacity was expressed as EC_50_, which is the concentration necessary for a 50% reduction in ABTS radicals. The ORAC values were calculated as the area under the curve (AUC) and expressed as μM of Trolox equivalent (TE).

#### 2.2.2. Cell Culture

Hs68 human dermal fibroblasts were purchased from the American Type Culture Collection (Manassas, VA, USA) and were cultured in monolayers at 37 °C in a 5% CO_2_ incubator in Dulbecco’s modified Eagle’s medium (DMEM) containing 10% fetal bovine serum.

#### 2.2.3. UVB Irradiation and Ce6-PEG-Cur-Mediated PDT

Hs68 cells (2 × 10^5^ cells/well) were seeded in a 6-well plate (Falcon, Corning, NY, USA) for 24 h, and then treated with 7 and 10 nM Ce6-PEG-Cur for 30 min at 37 °C in an atmosphere of 5% CO_2_ in the dark after UVB (200 mJ/cm^2^) irradiation. A fluorescent lamp emitting light at a wavelength of 312 nm (Model VL.215-LM, Vilber Lourmat, Eberhardzell, Germany) was used as the UVB source to induce photoaging. The Hs68 cells were then exposed to LED light (660 nm; 89 W/m^2^; 20 J/cm^2^; Donsung Lumex, Daegu, Korea) for 37 min 27 s and incubated for 3 h for PDT treatment. The Ce6-PEG-Cur-containing DMEM medium was then changed with serum-free DMEM medium and the Hs68 cells were incubated for 20 h (for MTT assay and Western blot) and 44 h (for collagen assay) in the dark.

#### 2.2.4. MTT Cell Viability Assay

Hs68 cell viability was measured using a 3-[4,5-dimethylthylthiazol-2-yl]-2,5 diphenyl tetrazolium bromide (MTT) assay. The cells were cultured in 24-well plates at a density of 3 × 10^4^ cells per well. The cells were then treated with varying concentrations of Ce6-PEG-Cur for 30 min in the dark. The Hs68 cells were then exposed to LED light (660 nm; 89 W/m^2^; 20 J/cm^2^) for 37 min 27 s and incubated for 3 h. The Ce6-PEG-Cur-containing DMEM medium was then changed with serum-free DMEM medium, and the Hs68 cells were incubated for 20 h. The cells were washed and treated with MTT, after which the plates were incubated at 37 °C in the dark for 2 h. After the formation of formazan, 100 μL of DMSO was added, and the absorbance was measured at 570 nm using a microtiter plate reader.

#### 2.2.5. Sircol Collagen Assay

The total soluble collagen in the Hs68 cell culture supernatant was quantified using the Sircol collagen assay (Biocolor, Belfast, UK). UVB-exposed Hs68 cells were incubated for another 44 h in the dark after Ce6-PEG-Cur-mediated PDT. Following this, 1 mL Sirius red dye, an anionic dye that reacts specifically with the basic side chain groups of collagens under the assay conditions, was added to 400 μL of cell culture medium supernatant and incubated with gentle rotation for 30 min at room temperature. After centrifugation, the pellet was washed with ice-cold acid-salt wash reagent, released in alkali reagent, and the absorbance at 570 nm was measured using an ELISA reader (Sunrise, Tecan, Männedorf, Switzerland). The amount of collagen was calculated based on a standard curve obtained with the standard bovine type Ⅰ collagen supplied with the kit.

#### 2.2.6. Western Blot Analysis

Whole cell lysates were sonicated in RIPA buffer (50 mM Tris/HCl, pH 7.4, 150 mM NaCl, 1 mM EDTA, 1 mM NaF, 1 mM Na_3_VO_4_, 1 mM PMSF, and 1% protease inhibitor cocktail). The lysed cells were centrifuged at 15,000 rpm for 50 min. The supernatants were decanted into new tubes, and the levels of protein in the supernatant were determined using a Bradford assay (Bio-Rad Laboratories, Inc. Hercules, CA). Each sample was prepared identically with reducing sample buffer (250 mM Tris-HCl, pH 6.8, 0.25% bromophenol blue, 50% glycerol, 10% SDS, 0.5 M DTT; Biosesang Inc., Seongnam, Korea) and separated by 9% SDS-PAGE gel at 90–110 V for 2 h. The separated proteins were transferred to polyvinylidene difluoride (PVDF) membranes (Bio-Rad Laboratories, Inc. Hercules, CA) at 0.4 A for 1 h. After transfer, the membrane was blocked with 5% bovine serum albumin (BSA) for 2 h with inversion. The blocked membrane was incubated for 18 h at 4 °C with primary antibodies against procollagen type Ⅰ (1:1000 dilution; Merck, Darmstadt, Germany), MMP-1, MMP-2, and MMP-9 (1:1000 dilution; Abcam, Cambridge, UK). After washing, the membranes were incubated with appropriate horseradish peroxidase-conjugated secondary antibodies for 1 h at room temperature. After repeated washing, the membrane was visualized with enhanced chemiluminescence (Wester Supernova, Cyanagen, Bologna, Italy) using a Chemidoc system (Sensi-Q2000 Chemidoc, Lugen Sci., Bucheon, Korea). For the in vivo Western blotting, the mouse skin was chopped and sonicated in RIPA buffer. The lysed tissues were prepared, and each sample contained 20 μg of total protein. The proteins were analyzed as described above for the in vitro Western blot assay. Images were captured, and the visualized areas were measured using ImageJ software to quantify the band intensities.

### 2.3. In Vivo Assay

#### 2.3.1. Experimental Animals

Five week old male SKH1 mice were purchased from Orient Bio Co., Ltd. (Seoul, Korea). Thirty hairless mice were assigned to six groups: (1) control group (n = 5); (2) UVB-irradiated (UVB) group (n = 5); (3) UVB-irradiated and LED (3 J/cm^2^)-irradiated (UVB-LED) group (n = 5); (4) Ce6-PEG-Cur 5 nM with UVB-LED group (n = 5); (5) Ce6-PEG-Cur 25 nM with UVB-LED group (n = 5); and (6) Ce6-PEG-Cur 50 nM with UVB-LED group (n = 5). The room temperature was maintained at 20 ± 2 °C, and the relative humidity was 60 ± 10%. The animals were maintained on a 12:12 h light:dark cycle. All animal procedures were approved by the Animal Research Ethics Committee of Soonchunhyang University (approval number: SCH18-0016).

#### 2.3.2. Measurement of Wrinkles Induced by UVB Irradiation

UVB irradiation was applied to the dorsal skin of the mice. The backs of the mice were exposed to UVB three times per week for 10 weeks, which is a modification of the method described by Kawada et al. (2010) [36]. Briefly, the initial dose was set at 36 mJ/cm^2^, which was subsequently increased to 54, 72, 108, and 144 mJ/cm^2^ at weekly intervals. Five weeks later, the mice were irradiated with UVB at 144 mJ/cm^2^ for 5 weeks. Three different concentrations of Ce6-PEG-Cur-mediated PDT (5, 25, and 50 nM) were performed after every UVB treatment. We used LED light (660 nm; 89 W/m^2^) for Ce6-PEG-Cur-mediated PDT at 3 J/cm^2^ for 5 min 37 s. The wrinkles on the backs of the mice were photographed using an Antera 3D camera (Miravex, Dublin, Ireland) after 10 weeks of Ce6-PEG-Cur-mediated PDT.

#### 2.3.3. Histological Analysis

After euthanasia with zoletil (Virbac Korea, Seoul, Korea), the skin samples were fixed in 10% formalin for 24 h and stained with H&E for skin layers and Masson’s trichrome for collagen fibers. The stained sections were analyzed using an Eclipse TE2000U inverted microscope with twin CCD cameras (Nikon, Tokyo, Japan).

### 2.4. Statistical Analysis

The data were statistically analyzed using the Statistical Package for Social Sciences (SPSS; version 20; SPSS Inc., Chicago, IL, USA). The data are representative of three independent experiments, and the values are expressed as the mean ± SD of the values from each group. Statistical significance was assessed using one-way analysis of variance (ANOVA) followed by Tukey’s post hoc test. The significance level was set at *p* < 0.05.

## 3. Results

### 3.1. Absorption Spectrum and Antioxidant Capacity of Ce6-PEG-Cur

**Figure 1:**
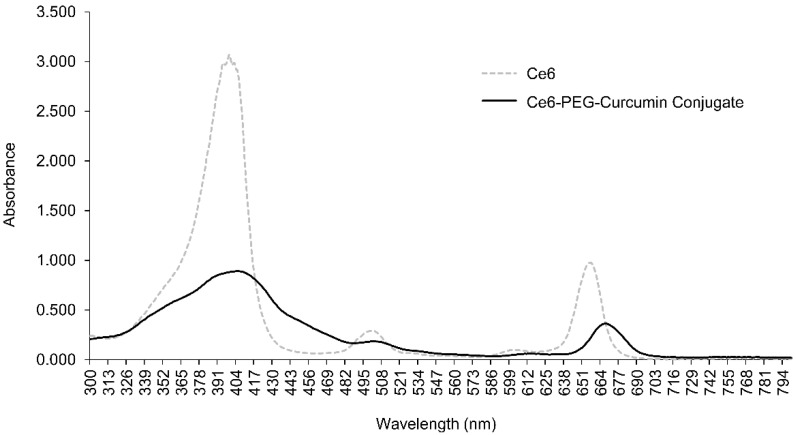
Absorption spectrum of Ce6-PEG-curcumin conjugate.

Figure 1 shows the absorption spectrum of the Ce6-PEG-curcumin conjugate. In the UV and visible spectral regions, the most intense bands appear at the boundary of the visible and UV regions of the spectrum at 404 nm (the Soret band) and at the boundary of the visible and IR regions of the spectrum at 660 nm. The distribution and intensity of the absorption maxima are approximately the same for Ce6 and the Ce6-PEG-curcumin conjugate.

The antioxidant activity of Ce6-PEG-Cur was measured using ABTS and ORAC assays, with and without light (Table 1). The EC_50_ values (effective concentration for 50% inhibition of the radicals) of scavenging ABTS radicals for Ce6-PEG-Cur without and with LED light irradiation were 18.77 ± 4.55 and 23.31 ± 9.82 μM, respectively. The antioxidant activity (measured by ORAC assay) of Ce6-PEG-Cur without and with LED light was 34.48 and 27.40 μM TE scavenging capacity, respectively. The antioxidative capacity was slightly reduced by LED light application in both the ABTS and ORAC assays.

### 3.2. The Effects of Ce6-PEG-Cur-Mediated PDT on the Viability of Fibroblasts

**Figure 2:**
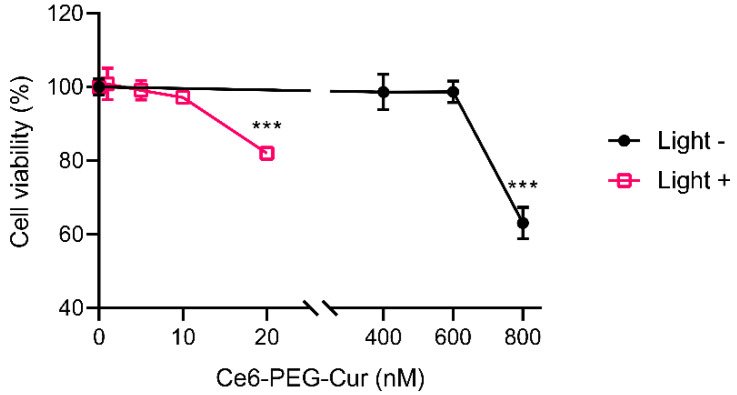
Cell viability of Ce6-PEG-curcumin with and without LED irradiation, measured by MTT assay. Results are expressed as mean ± SD. *** *p* < 0.001 vs. control.

The viability of dermal fibroblasts treated with various concentrations of Ce6-PEG-Cur with and without LED light was examined using the MTT assay. The resulting survival curve shown in Figure 2 indicates that non-irradiated control cells were considered 100% viable at up to 600 nM Ce6-PEG-Cur. However, after irradiation with LED light, cell viability was significantly reduced. The maximum concentration of Ce6-PEG-Cur without cell damage was 10 nM Ce6-PEG-Cur. Irradiation with LED alone did not show any significant effect on the cell viability (data not shown).

### 3.3. Inhibitory Effect of Ce6-PEG-Cur-Mediated PDT on UVB-Induced MMP and Procollagen Expression

**Figure 3:**
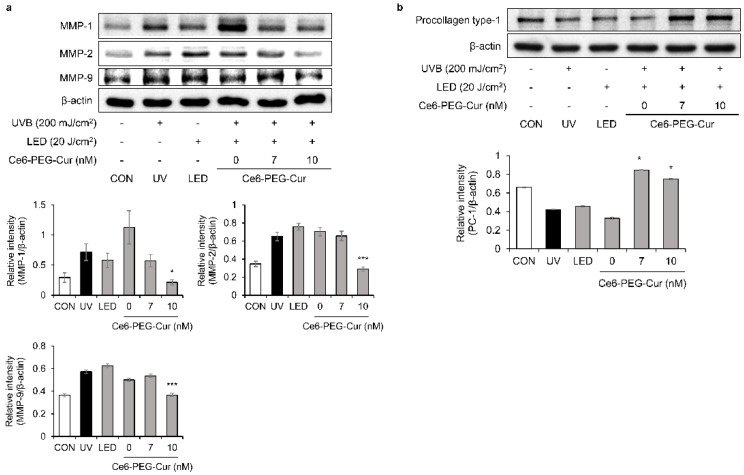
The effect of Ce6-PEG-curcumin-mediated PDT on MMPs and procollagen type Ⅰ in UVB-irradiated Hs68 cells. The effect of Ce6-PEG-curcumin-mediated PDT on (**a**) the expression of MMP-1, MMP-2, and MMP-9 protein and (**b**) the expression of procollagen type Ⅰ in UVB-irradiated Hs68 cells. Results are expressed as mean ± SD. * *p* < 0.05 and *** *p* < 0.001 vs. UVB-irradiated control.

The effect of Ce6-PEG-Cur-mediated PDT on the expression of MMP-1, MMP-2, and MMP-9 proteins was measured in UVB-irradiated fibroblast cells using Western blotting (Figure 3a). UVB-induced MMP-1, MMP-2, and MMP-9 protein expression was significantly decreased in a dose-dependent manner by Ce6-PEG-Cur-mediated PDT. At 10 nM Ce6-PEG-Cur, the expression levels of MMP-1, 2, and 9 were reduced to that of the control. Notably, type I procollagen protein expression was significantly increased by 7 nM and 10 nM of Ce6-PEG-Cur-mediated PDT compared to UVB irradiation alone (Figure 3b). The results show that UVB-induced MMP expression was reduced by Ce6-PEG-Cur-mediated PDT, whereas type I procollagen protein expression was enhanced.

### 3.4. Inhibitory Effects of Ce6-PEG-Cur-Mediated PDT on the Expressions of NF-kB-Dependent Proteins

**Figure 4:**
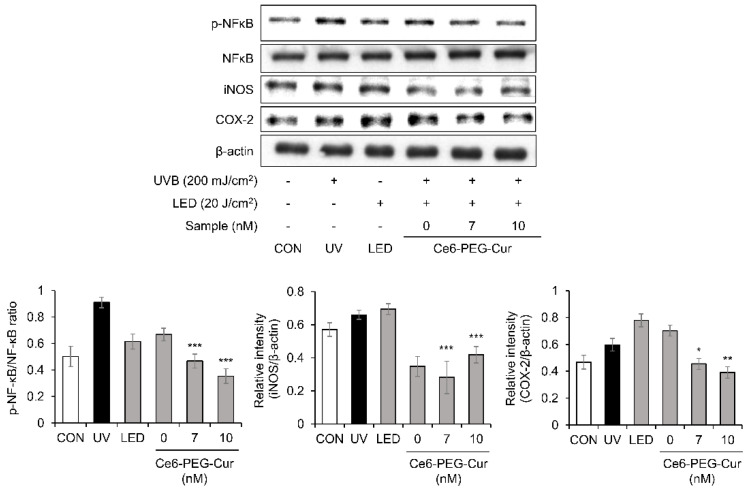
Inhibition of UVB-induced expression of p-NF-κB, iNOS and COX-2 by Ce6-PEG-curcumin-mediated PDT. Results are expressed as mean ± SD. * *p* < 0.05, ** *p* < 0.01 and *** *p* < 0.001 vs. UVB-irradiated control.

The effect of Ce6-PEG-Cur-mediated PDT on the expression of NF-κB protein was examined using Western blotting (Figure 4). The expression of phosphorylated NF-κB was significantly increased in UVB-irradiated fibroblasts. However, this increase in expression was attenuated by Ce6-PEG-Cur-mediated PDT. The expression of NF-κB induced by UVB is known to be associated with inflammatory enzymes, including COX-2 and iNOS. UVB irradiation induced the expression of the COX-2 and iNOS proteins in fibroblasts; however, Ce6-PEG-Cur-mediated PDT significantly decreased the levels of both proteins. These results show that Ce6-PEG-Cur-mediated PDT regulates NF-κB activation and the expression of the inflammatory enzymes iNOS and COX-2.

### 3.5. Effects of Ce6-PEG-Cur-Mediated PDT on UVB-Induced Activation of p38 MAP Kinase

**Figure 5:**
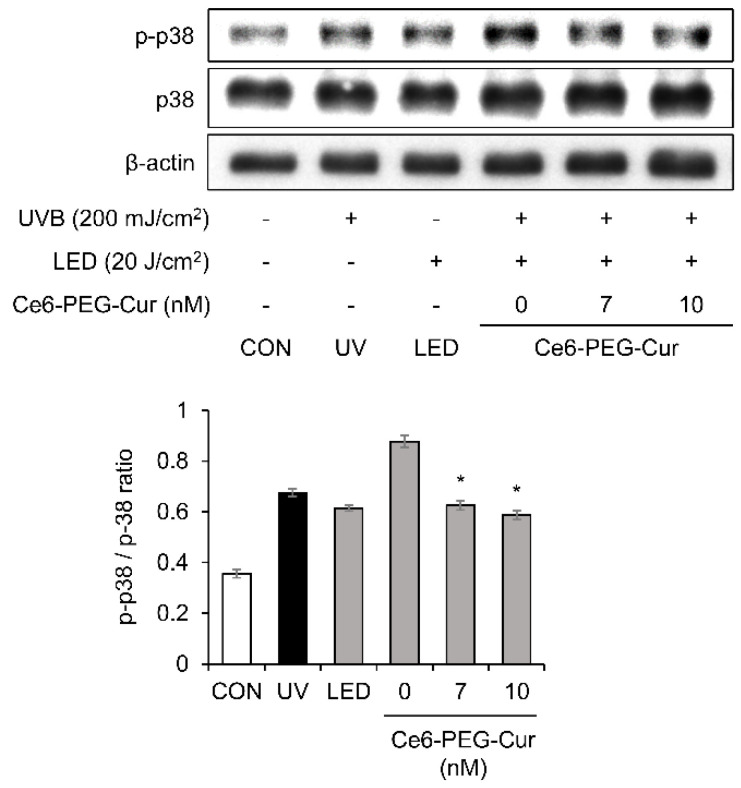
The effect of Ce6-PEG-curcumin-mediated PDT on the phosphorylation p38 MAPK and c-Jun expression in UVB-irradiated HS68 cells. Results are expressed as mean ± SD. * *p* < 0.05 vs. UVB-irradiated control.

UVB exposure significantly increased the expression of phosphorylated p38 MAPK in the fibroblasts (Figure 5). However, phosphorylation of p38 MAPK was attenuated by Ce6-PEG-Cur-mediated PDT treatment.

### 3.6. Effect of Ce6-PEG-Cur-Mediated PDT on In Vitro Collagen Production

**Figure 6:**
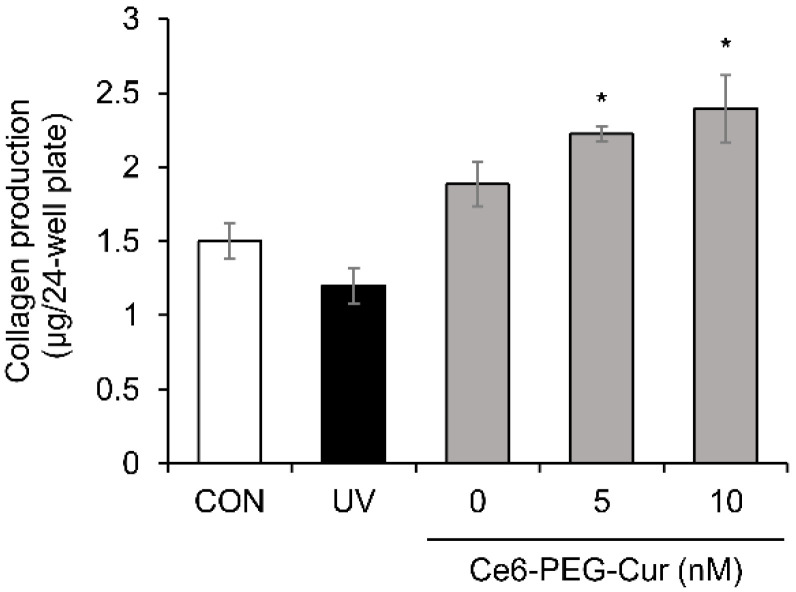
The release of soluble collagen into the conditioned medium of Hs68 fibroblast cells supplied with Ce6-PEG-curcumin-mediated PDT was determined by Sircol collagen assay. Results are expressed as mean ± SD. * *p* < 0.05 vs. UVB-irradiated control.

The production of soluble type Ⅰ procollagen was examined using the Sircol collagen assay (Figure 6). UVB-induced collagen levels were reduced by 20% compared to the non-irradiated control. However, an approximately two-fold increase in type Ⅰ procollagen level was found in the 10 nM Ce6-PEG-Cur-mediated PDT group compared to the UVB-irradiated group.

### 3.7. In Vivo Expression of Type Ⅰ Procollagen and MMP Proteins in the Skin of Mice Treated with UVB-Irradiated Mice

**Figure 7:**
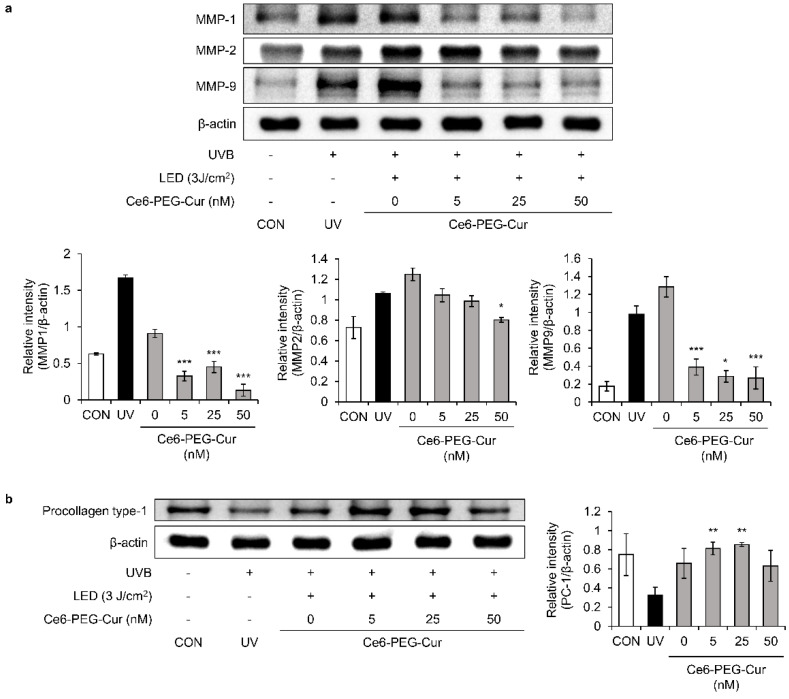
The effect of Ce6-PEG-curcumin-mediated PDT on (**a**) the MMP-1, MMP-2, and MMP-9 expression and (**b**) procollagen type Ⅰ levels in the dorsal skin of UVB-irradiated mice. Results are expressed as mean ± SD. * *p* < 0.05, ** *p* < 0.01, and *** *p* < 0.001 vs. UVB-irradiated control.

The in vivo effect of Ce6-PEG-Cur-mediated PDT on the expression of the MMP-1, MMP-2, and MMP-9 proteins was examined in SKH-1 hairless mouse skin using Western blotting (Figure 7a). The expression of MMP-1, MMP-2, and MMP-9 in mice exposed to UVB was significantly increased. However, this increase was dramatically attenuated by Ce6-PEG-Cur-mediated PDT. In particular, the expression of MMP-1 and MMP-9 was greatly decreased compared to the UVB-irradiated mice. We further investigated whether Ce6-PEG-Cur-mediated PDT could induce the expression of type Ⅰ procollagen in an in vivo system using Western blotting. Similar to the results of the in vitro collagen assay, the expression of type Ⅰ pro-collagen was dramatically increased over the UVB untreated control following Ce6-PEG-Cur-mediated PDT (Figure 7b).

The results confirmed that Ce6-PEG-Cur-mediated PDT significantly reduced UVB-induced MMP expression and enhanced type Ⅰ procollagen expression in an in vivo system.

### 3.8. Suppressive Effect of Ce6-PEG-Cur-Mediated PDT on Wrinkle Formation in UVB-Irradiated Hairless Mice

**Figure 8:**
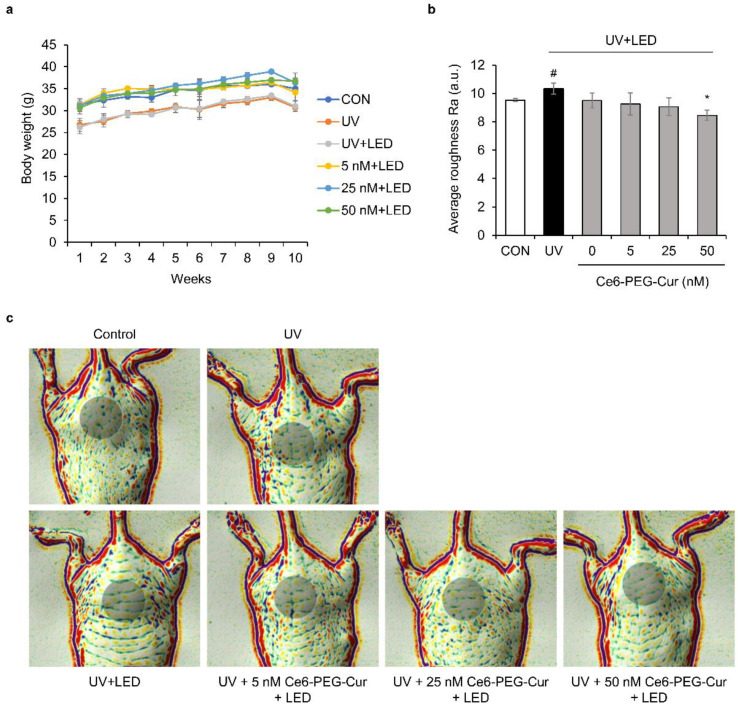
(**a**) Change in the body weight of SKH-1 hairless mice over 10 weeks of Ce6-PEG-curcumin-mediated PDT treatment. (**b**,**c**) The evaluation of SKH-1 hairless mice using Antera 3D after 10 weeks of Ce6-PEG-curcumin-mediated PDT. The average roughness Ra of dorsal skin was evaluated in the shaded circle area. Results are expressed as mean ± SD. * *p* < 0.05 vs. UVB-irradiated control. # *p* < 0.05 vs. control.

The in vivo efficacy of Ce6-PEG-Cur-mediated PDT was investigated in UVB-induced wrinkle formation in hairless mice (Figure 8). Body weight and food intake were not significantly different between the experimental groups during the 10 week experimental period (Figure 8a). The effect of Ce6-PEG-Cur-mediated PDT on wrinkle formation by UVB irradiation was investigated using an Antera 3D camera in the dorsal skin of mice. As shown in Figure 8b,c, the dorsal skin of UVB-irradiated mice was rougher than that of the control mice. However, the roughness was significantly reduced following 50 nM Ce6-PEG-Cur-mediated PDT treatment compared with that of mice exposed to UVB irradiation alone.

### 3.9. Effects of Ce6-PEG-Cur-Mediated PDT on the Skin Thickness and Collagen in UVB-Irradiated Hairless Mice

**Figure 9:**
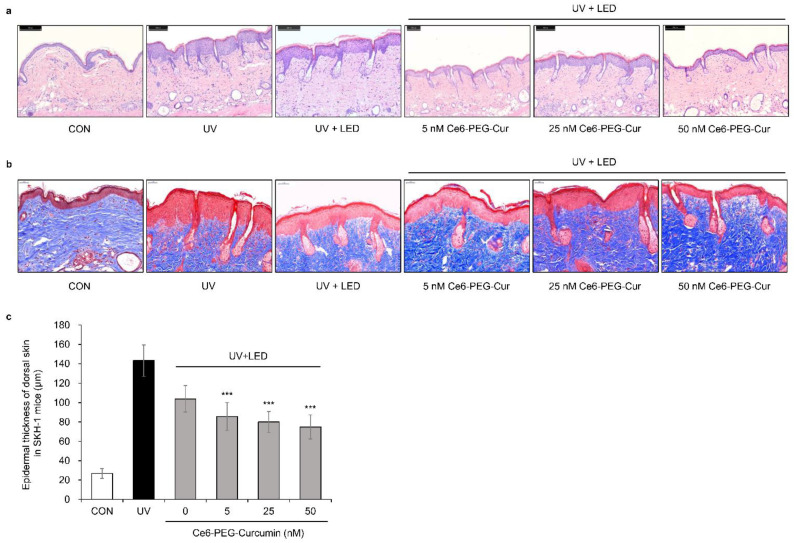
The effect of Ce6-PEG-curcumin-mediated PDT on the epidermal thickness and density of collagen fibers in the dorsal skin of UVB-irradiated mice. (**a**) H&E staining. Scale bar = 200 μm. (**b**) Masson’s trichrome staining. Scale bar = 50 μm. (**c**) Histogram of hematoxylin and eosin staining. Results are expressed as mean ± SD (n = 5). *** *p* < 0.001 vs. UVB-irradiated control.

The effect of Ce6-PEG-Cur-mediated PDT on UVB-irradiated dorsal skin was investigated histologically (Figure 9). The dorsal skin of SKH-1 hairless mice in each group was stained with hematoxylin and eosin (H&E) (Figure 9a) and Masson’s trichrome (Figure 9b) to investigate the histological changes in the dermal layer and collagen fibers. H&E staining revealed that the UVB-irradiated mice showed an observable increase in epidermal thickness compared to the control group. However, UVB-irradiated mice treated with Ce6-PEG-Cur-mediated PDT showed a notable recovery from the pathological changes compared to UVB irradiation alone (Figure 9c). Masson’s staining revealed that collagen fibers were damaged upon exposure to UVB irradiation. However, PDT suppressed the UVB irradiation-induced loss of collagen fibers. These results suggest that Ce6-PEG-Cur-mediated PDT can attenuate UVB-irradiation-induced skin thickening and collagen fiber loss, leading to protection against UVB-induced skin damage.

## 4. Discussion

UVB from sunlight can induce ROS formation in the skin, leading to the upregulation of MMP expression and wrinkle formation [19]. UVB exposure leads to the activation of the transcription factors AP-1 and NF-κB, which stimulate MMP genes in the skin [37,38]. Moreover, the increased ROS in UVB-irradiated skin stimulates protein kinase cascades, including MAPKs [39]. MAPK proteins, such as p38, JNK, and ERK, activate the dimers of Jun and Fos, ultimately inducing MMP expression [40,41].

In this study, Ce6-PEG-Cur-mediated PDT reduced the expression of the MMP-1, MMP-2, and MMP-9 proteins in UVB-irradiated Hs68 cells (Figure 3) and the dorsal skin of UVB-irradiated SKH-1 mice (Figure 7). The efficacy of Ce6-PEG-Cur-mediated PDT in reducing UVB-induced MMP expression might be exerted via a reduction in MAPK/NF-κB signaling. Ce6-PEG-Cur-mediated PDT downregulated p-p38 and NF-κB signals (Figure 4 and Figure 5) in UVB-irradiated Hs68 cells. MMPs lead to cross-linked collagen fragmentation and a breakdown of the dermal ECM structure [42]. MMP-1 initiates the cleavage of type Ⅰ and Ⅲ collagens in the skin, allowing the cleaved collagen to be further degraded by MMP-3 and MMP-9 [43,44]. Thus, topical MMP activation may be an effective target for preventing UVB-induced wrinkle formation.

Ce6-PEG-Cur-mediated PDT increased the level of procollagen type Ⅰ in UVB-irradiated Hs68 cells (Figure 3b) and procollagen type Ⅰ in the dorsal skin of UVB-irradiated SKH-1 mice (Figure 7b). These results suggest that the UVB-induced degradation of collagen was counteracted by Ce6-PEG-Cur-mediated PDT by reducing the expression of MMP-1, MMP-2, and MMP-9. Additionally, Ce6-PEG-Cur-mediated PDT seems to increase collagen synthesis in both Hs68 cells and SKH-1 mice, because the collagen production in Hs68 cells increased and the collagen density of UVB-irradiated SKH-1 mouse skin was elevated. Moreover, UVB-induced dorsal roughness was significantly reduced following 50 nM Ce6-PEG-Cur-mediated PDT treatment compared with that in mice exposed to UVB irradiation alone.

The antioxidative effect of Ce6-PEG-Cur in the presence of PDT might contribute to the in vitro and in vivo anti-photoaging effects demonstrated in this study. In conclusion, PDT using Ce6-PEG-Cur could be an effective therapeutic agent for the rejuvenation of photoaged skin.

## Figures and Tables

**Table 1:** Antioxidant activity of Ce6-PEG-curcumin with and without light irradiation determined by ABTS and ORAC assay.

Photosensitizer	μM	ABTS	ORAC (μM TE)
		EC_50_ (μM)	
Ce6-PEG-Curcumin/Light (−)	1	18.77 ± 4.55	9.25
	10	-	34.48
Ce6-PEG-Curcumin/Light (+)	1	23.31 ± 9.82	1.40
	10	-	27.40

## Data Availability

Not applicable.
